# Adaptation to full weight‐bearing following disuse in rats: The impact of biological sex on musculoskeletal recovery

**DOI:** 10.14814/phy2.15938

**Published:** 2024-02-21

**Authors:** Margot Issertine, Megan E. Rosa‐Calwell, Dong‐Min Sung, Mary L. Bouxsein, Seward B. Rutkove, Marie Mortreux

**Affiliations:** ^1^ Department of Neurology Beth Israel Deaconess Medical Center Boston Massachusetts USA; ^2^ Harvard Medical School Boston Massachusetts USA; ^3^ Department of Orthopedic Surgery Beth Israel Deaconess Medical Center, Center for Advanced Orthopaedic Studies Boston Massachusetts USA; ^4^ Department of Nutrition University of Rhode Island Kingston Rhode Island USA

**Keywords:** bone, gravity, muscle, musculoskeletal, rats, reloading, sex, unloading

## Abstract

With the technological advances made to expand space exploration, astronauts will spend extended amounts of time in space before returning to Earth. This situation of unloading and reloading influences human physiology, and readaptation to full weight‐bearing may significantly impact astronauts' health. On Earth, similar situations can be observed in patients who are bedridden or suffer from sport‐related injuries. However, our knowledge of male physiology far exceeds our knowledge of female's, which creates an important gap that needs to be addressed to understand the sex‐based differences regarding musculoskeletal adaptation to unloading and reloading, necessary to preserve health of both sexes. Using a ground‐based model of total unloading for 14 days and reloading at full weight‐bearing for 7 days rats, we aimed to compare the musculoskeletal adaptations between males and females. Our results reveal the existence of significant differences. Indeed, males experienced bone loss both during the unloading and the reloading period while females did not. During simulated microgravity, males and females showed comparable muscle deconditioning with a significant decline in rear paw grip strength. However, after 7 days of recovery, muscle strength improved. Additionally, sex‐based differences in myofiber size existing at baseline are significantly reduced or eliminated following unloading and recovery.

## Graphical abstract

Schematic representation of the experimental design and outcomes. It highlights changes in grip strength and bone mineral density during unloading and recovery at normal weight‐bearing in males and females.

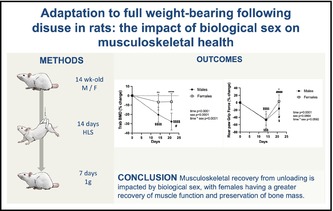

## 1 INTRODUCTION

Extended time spent in microgravity (either real or simulated) is associated with severe muscle deconditioning (Fitts et al., 2000), bone loss (Gabel et al., 2022), and cardiovascular alterations (Shen & Frishman, 2019), despite the use of countermeasures such as exercise training (Petersen et al., 2016). With the Artemis missions aiming to establish a sustained presence on the lunar surface and prepare for the long journey to Mars (NASA, 2017; Smith et al., 2020), astronauts will have to face severe deconditioning before re‐adjusting to Earth's gravity. Previous studies have established that while astronauts usually experience a 1%–1.5% bone loss per month (Vico et al., 2017) during their mission, they also fail to recover their normal bone density long after return (Dana Carpenter et al., 2010; Orwoll et al., 2013). While this represents an important health risk increase for astronauts (Droppert, 1990; Edgerton & Roy, 1994; Gabel et al., 2022; Lambrecht et al., 2017; Pool‐Goudzwaard et al., 2015), the succession of unloading and reloading periods is not solely experienced by this population. Indeed, mechanical unloading is also experienced on Earth, often as a result of bed rest, immobilization, or injury. These conditions lead to substantial muscle atrophy and reduced force generation capabilities (Dos Santos et al., 2016; Krasnoff & Painter, 1999; Sargeant et al., 1977), often more so in women than men (Abadi et al., 2009; Yasuda et al., 2005). Injured athletes who have been immobilized or have suffered from anterior cruciate ligament (ACL) injury still show muscle atrophy and weakness more than a year after intervention and return to full weight‐bearing (Grimby et al., 1980; Hortobágyi et al., 2000), which can result in reduced physical performance and increase the risk of future injury (Ingersoll et al., 2008; Mendias et al., 2013). However, studies comparing post‐injury capability in adults of both sexes have yielded conflicting results (Gianakos et al., 2022; Maguire et al., 2021; Sutton & Bullock, 2013), and rehabilitation protocols remain identical for both sexes (Adams et al., 2012; Maguire et al., 2021).

Similarly, each day spent on bed rest in the intensive care unit (ICU) decreases muscle strength by 3%–11% when adjusted for other risk factors (Fan et al., 2014), and frequently results in persistent muscle weakness which impacts both physical function and quality of life (Fan et al., 2014; Parry & Puthucheary, 2015), commensurate with patient's age (Hashem et al., 2016). However, few of these studies have examined the impact of biological sex on muscle weakness and physical performance after the disuse period. Although previous rodent studies have investigated muscle recovery after unloading (Mozdziak et al., 2000; Oishi et al., 2008), sex differences have not been explored.

The purpose of our study was to compare how male and female rats recover from unloading‐induced musculoskeletal deconditioning (using the hindlimb suspension model, HLS) after returning to full weight‐bearing. Our results demonstrate the existence of sex‐based differences in musculoskeletal adaptation to both unloading and reloading. These results highlight important differences that should be considered during musculoskeletal recovery, and could help identify new considerations for strategies aiming to preserve musculoskeletal health, both on Earth and in space.

## 2 MATERIALS AND METHODS

### 2.1 Animals

Twenty‐one (Ingersoll et al., 2008) adult Wistar rats (10 males and 11 females) were obtained at 13 weeks of age (Charles River Laboratories, Wilmington, MA), and baseline assessments were performed 1 week later. Prior to baseline testing, animals were individually housed and placed in custom cages and allowed to acclimate for 24‐48 h. These cages, described elsewhere (Mortreux et al., 2018; Mortreux & Rosa‐Caldwell, 2020), are used to perform hindlimb suspension (HLS) and to house animals during full weight‐bearing recovery. Rats were placed in HLS for 14 days using a pelvic harness as previously described (Mortreux et al., 2019, 2021), before being allowed to return to full weight‐bearing for 7 days.

Water and rat chow were provided ad libitum, and food intake was recorded (LabDiet Formulab Diet Rodent Chow, Cat #5008i). Daily checks were performed to assess animals' well‐being and monitor for any signs of pain or discomfort (e.g., porphyric staining around the eyes, poor grooming, prostration and hair loss), appropriate harness fitting, and the ability to walk unimpeded across the cage.

### 2.2 Grip strength testing

Grip strength testing was obtained during baseline testing and weekly thereafter. Rats' rear paws were placed on a 50 N capacity digital grip force meter (Chatillon, Largo, FL, USA) and gently pulled backward until the animal released its grip from the pull bar. The same protocol was used for the front paws. Three tests were performed with a short latency period, and the peak force generated during each test was recorded and averaged.

### 2.3 Nerve‐stimulated force production

Force production was measured at baseline, after 14 days of HLS (Day 14, R + 0), and after 7 days of reloading (Day 21, R + 7). Animals were anesthetized using vaporized isoflurane (1.5%–3.5%) + O_2_. Rats were placed on a force plate (Dual Mode Muscle Lever System, Aurora Scientific, Aurora, ON, Canada), with the left foot securely taped to the apparatus. Monopolar electrodes (28G, Natus Medical Incorporated, Pleasanton, CA, USA) were placed subcutaneously to deliver a supramaximal, tetanic stimulation at 200 Hz for 200 ms of the peroneal nerve at the fibular head (foot dorsiflexion) or a stimulation of the tibial nerve at the popliteal fossa (foot plantar flexion). The maximal torque response was recorded, and the area under the curve (AUC) was calculated.

### 2.4 Fatigue protocol

Anesthetized animals connected to the footplate apparatus were subjected to a fatigue plantar flexion protocol, where they received a 120 s stimulation train (frequency: 40 Hz, pulse duration: 200 ms, pulse delay: 800 ms, repetitions: 120) as described elsewhere (Bagni et al., 2019; Fuglevand et al., 1999; Gineste et al., 2020). The maximal force produced during the protocol was recorded, and the AUC of the force curve was calculated.

### 2.5 In vivo skeletal assessments at the tibial diaphysis

At baseline (Day 0), immediately after unloading (Day 14, R + 0) and after reloading (Day 21, R + 7), we assessed total and trabecular volumetric bone mineral density (vBMD) at the proximal tibia and cortical vBMD at the tibial mid‐diaphysis, using in vivo peripheral quantitative computed tomography (pQCT, Stratec, XCT Research SA+, Pforzheim, Germany), as previously described (Ko et al., 2020). Daily calibration of the machine was performed using a manufacturer‐supplied hydroxyapatite phantom. Scans were performed in anesthetized animals using a voxel size of 100 μm and a scanning beam thickness of 500 μm. Transverse images of the right tibia were acquired at 5.0, 5.5, 6.0 mm (for total and trabecular vBMD), and at 20 and 20.5 mm (for cortical vBMD) distal to the proximal tibial plateau. Scanned slices were analyzed using Stratec software (Stratec XCT Analysis System, v6.0, Norland Corp., Fort Atkinson, WI), and a standardized analysis to determine cortical (contour mode 1, peel mode 2, outer and inner threshold of 0.650 g/cm^3^) and trabecular (contour mode 3, peel mode 4, outer threshold of 0.450 g/cm^3^, inner threshold of 0.800 g/cm^3^) vBMD.

### 2.6 Estrous cycle monitoring

Estrous cycle monitoring was performed daily around 8:00 am, as previously described (Caligioni, 2009; Rosa‐Caldwell et al., 2021). Briefly, a pipette tip containing 50 μL of ddH_2_O was gently inserted at the vaginal opening. The cavity was flushed 2–3 times to collect vaginal wall cells. The sample was then placed on a microscope slide and allowed to dry, before being stained using 0.1% Crystal Violet solution (Sigma‐Aldrich, C0775‐25G, St Louis, MO). After being rinsed with water, slides were visualized using a light microscope (×40 magnification) and the estrous cycle phase was determined using cellular anatomical landmarks by a single researcher who was blinded to experimental conditions. Proestrus (P) and estrus (E) were considered “high hormonal states” and characterized by a majority of nucleated (P) or of non‐nucleated cornified (E) epithelial cells. Metestrus (M) and diestrus (D) were considered “low hormonal states” and characterized by the presence of leukocytes, nucleated epithelial cells, and cornified cells (M), or by the presence of leukocytes (D), respectively.

### 2.7 Tissue collection

Animals were euthanized at the end of the study by CO_2_ inhalation, according to IACUC guideline and in agreement with the recommended practices. Left hind limb muscles (i.e., gastrocnemius, soleus, extensor digitorum longus (EDL), and tibialis anterior (TA)) were immediately harvested, and the wet mass of each muscle was determined using a precision analytical balance. The muscles were then placed in 10% neutral buffered formalin and fixed for 48 h at 4°C, before being washed and stored in phosphate buffered saline (PBS) for histological analysis.

### 2.8 Muscle histomorphometry

Soleus muscles were embedded in paraffin blocks, sectioned into 10 μm slices and triple immunofluorescence staining was performed using and anti‐slow skeletal myosin heavy chain antibody (ab11083, Abcam, Cambridge, MA, USA), anti‐fast skeletal myosin heavy chain antibody (ab91506, Abcam, Cambridge, MA, USA), and anti‐wheat germ agglutinin (W6748, Thermofisher Scientific, Waltham, MA, USA) as previously described (Mortreux et al., 2021). Stained slides were subsequently imaged at 20× using a Zeiss Axio Imager M1 epifluorescence microscope, and myofiber cross‐sectional area (CSA) was measured using the muscle morphometry plug‐in (Anthony Sinadinos using Eclipse IDE) and FIJI (FIJI, ImageJ, NIH), with experimenters being blinded during both image acquisition and image analysis. CSA data from normally loaded controls (NL R + 0) and hindlimb suspended controls (HLS R + 0) were obtained from animals whose results were the object of another publication (Mortreux et al., 2021); however, no data were duplicated.

### 2.9 Statistical analyses

Data were analyzed with GraphPad Prism v10.1.1 (GraphPad Software, La Jolla, CA). To compare the responses in animals of both sexes, we used the percentage change from baseline for each animal for in vivo assessments. After performing normality tests, longitudinal data were analyzed using two‐way repeated measures ANOVA (effects of time and sex), followed by Tukey's or Sidak's post hoc multiple comparisons test. Terminal assessments were analyzed using unpaired Student's *t*‐tests or two‐way ordinary ANOVA followed by Tukey's post hoc test. Results are represented as mean ± SD unless specified otherwise and were considered significant when *p* < 0.05.

## 3 RESULTS

Fourteen days of mechanical unloading using hindlimb suspension (HLS) resulted in significant body weight loss in males and females (Table 1). After 7 days of reloading at full weight‐bearing, females recovered all the weight lost during unloading but males did not (*p* < 0.01). However, these changes were not attributable to differences in food consumption between the disuse and recovery periods. After unloading, females had a decrease in non‐fasted blood glucose levels compared to baseline, despite remaining within normal values, while males did not experience any variation (Table 1).

**TABLE 1:** Animals' body weight, blood glucose and average weekly food intake during disuse (Day 0–Day 14) and reloading (Day 14–Day 21).

	Males (*n* = 10)			Females (*n* = 11)		
	Day 0	Day 14	Day 21	Day 0	Day 14	Day 21
Body weight (g)	436.16 ± 27.39	410.27 ± 29.81**	416.14 ± 29.97**	263.29 ± 24.85	251.15 ± 23.21*	263.62 ± 21.76^$^
Body weight (% change from Day 0)	8e^−9^ ± 6.29	−5.93 ± 3.74*	−4.59 ± 3.42^$^	−1e^−8^ ± 9.29	−4.52 ± 3.98	+0.37 ± 5.67^$$^
Average weekly food intake (g)	N/A	172.47 ± 9.92	168.04 ± 14.41	N/A	125.55 ± 8.06	130.60 ± 20.16
Blood glucose (mg/dL)	109.5 ± 13.5	103.1 ± 14.1	107.2 ± 10.7	99.0 ± 9.8	86.0 ± 11.6*	92.0 ± 8.7

*Note*: Average weekly food intake for Day 14 represents the average value from Day 0 to Day 14. Average weekly food intake for Day 21 represents the average value from Day 14 to Day 21. N/A, Not applicable. Results are displayed for males (*n* = 10) and females (*n* = 11) as mean ± SD. *, **: *p* < 0.05, *p* < 0.01 versus Day 0; $, $$: *p* < 0.05, *p* < 0.01 versus Day 14.

### 3.1 Trabecular loss is more pronounced in males and persists during reloading

**FIGURE 1:**
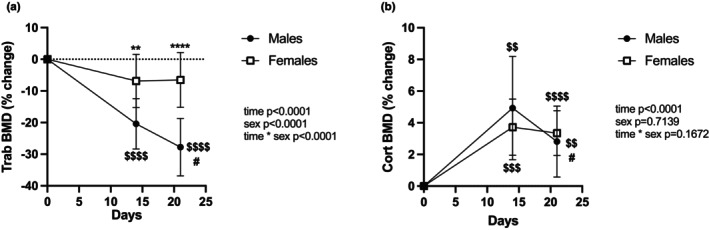
Skeletal changes at the tibial diaphysis. Change in trabecular bone mineral density (Trab. BMD) (a) and cortical bone mineral density (Cort. BMD) (b), measured weekly using pQCT in male and female rats. *N* = 10 for males, *n* = 11 for females. The results of the two‐way RM ANOVA are displayed in Table S1, and post hoc test results are represented as **, ****: *p* < 0.01, *p* < 0.0001 versus males; $$, $$$, $$$$: *p* < 0.01, *p* < 0.001, *p* < 0.0001 versus Day 0; #: *p* < 0.05, versus Day 14. Experiment Days 1–14 were spent in HLS, and Days 15–21 were spent at 1 g (normal loading).

HLS led to a greater decline in tibial trabecular bone mineral density (Trab.BMD) in males (−20.4% ± 2.5%) than females (−6.8% ± 2.5%, Figure 1a). After being reloaded for 7 days, Trab.BMD remained stable in females and did not differ from baseline. In contrast, males displayed further reduction in Trab.BMD, with values decreasing to 27.8% ± 2.9% lower than baseline, significantly lower than the values obtained at the end of the unloading period (*p* = 0.041).

Cortical bone mineral density (Cort.BMD) at the tibial diaphysis increased during both the unloading and reloading periods in animals of both sexes (*p* < 0.0001), with no differences between groups (*p* = 0.71, Figure 1b). However, after 7 days of reloading, males displayed lower values than after HLS (*p* = 0.019) while females did not.

### 3.2 Reloading increases force production in females more than males

**FIGURE 2:**
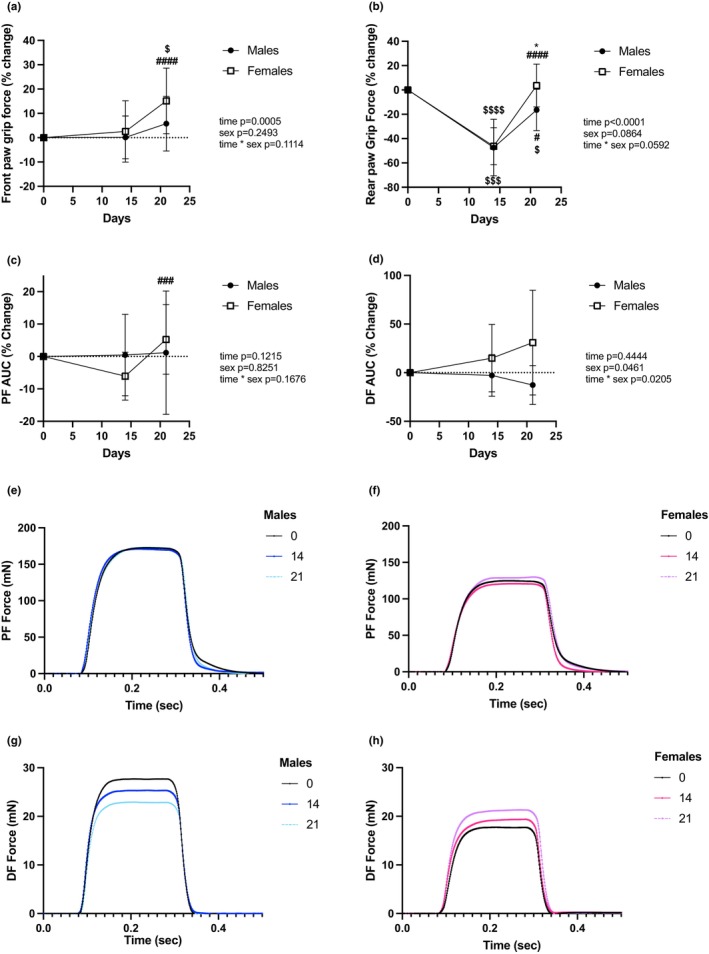
Functional muscle assessments. Change in front (a) and rear (b) paw grip force, change in the area under the curve (AUC) during plantar flexion, labeled as PF (c), and dorsiflexion, labeled as DF (d). Representative force curve for plantar flexion (e, g) and for dorsiflexion (f, h) are plotted. Males' data are shown in blue, and females' data are shown in pink. *N* = 10 for males, *n* = 11 for females. The results of the two‐way RM ANOVA are displayed in Table S1, and post hoc test results are represented as *: *p* < 0.05 versus males; $, $$$$: *p* < 0.05, *p* < 0.0001 versus Day 0; #, ###, ####: *p* < 0.05, *p* < 0.001, *p* < 0.0001 versus Day 14. Experiment Days 1–14 were spent in HLS, and Days 15–21 were spent at 1 g (normal loading).

Front paw grip strength was not impacted during HLS but increased during mechanical reloading (Figure 2a). Female rats showed greater improvement in grip force than males with a 15.1% ± 2.9% increase from baseline after the reloading period. On the other hand, rear paw grip strength significantly decreased after HLS in both sexes (Figure 2b). Both groups were stronger after 7d of recovery, with females exhibiting a greater increase in grip force (*p* < 0.0001).

Nerve‐stimulated force production measured during plantar flexion remained stable throughout disuse and recovery in males (Figure 2c), while females showed a non‐significant decline after the HLS period. During foot dorsiflexion measurements, we observed opposing profiles in males and females (Figure 2d). Indeed, males produced less force over time, with a minimum reached after the end of the reloading phase (−12.7% ± 9.2%), while females increased force production after the reloading period (+28.7 ± 8.8), although no group achieved statistical significance. These changes are most clearly visible on the representative tetanic curves provided for males (Figure 2e,g) and females (Figure 2f,h), respectively.

### 3.3 Muscle endurance is less impacted in females compared to males

**FIGURE 3:**
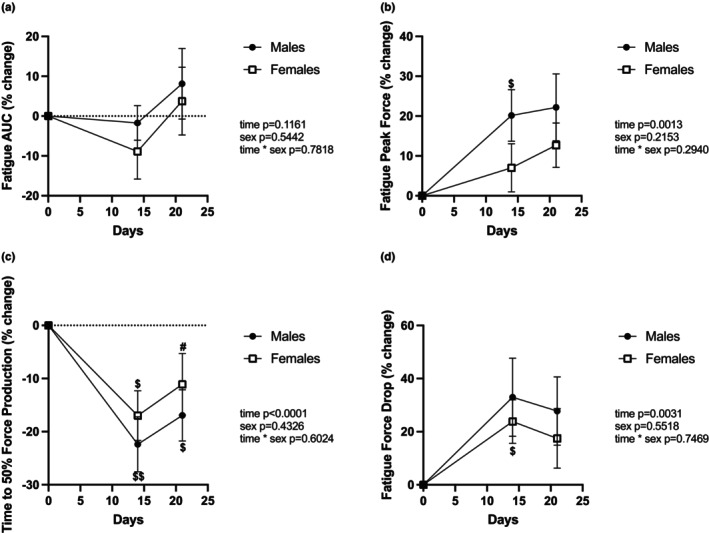
Muscle resistance to a fatigue protocol. Change in the area under the curve (AUC) during the fatigue protocol (a), change in fatigue peak force (b), change in the time to decrease force production by 50% (c), and change in fatigue force drop (d). *N* = 10 for males, *n* = 11 for females. The results of the two‐way RM ANOVA are displayed in Table S1, and post hoc test results are represented as $, $$: *p* < 0.05, *p* < 0.01 versus Day 0; #: *p* < 0.05, versus Day 14. Experiment Days 1–14 were spent in HLS, and Days 15–21 were spent at 1 g (normal loading).

Fatigability was assessed in the posterior compartment (i.e., gastrocnemius and soleus) during a foot plantar flexion (Figure 3). Overall, HLS and mechanical reloading did not appear to impact the development of fatigue in either sex (Figure 3a); however, we observed a constant increase in the peak force recorded (effect of time *p* = 0.0013, Figure 3b). After unloading, animals of both sexes fatigued more quickly than at baseline (Figure 3c), but females showed improved fatigue resistance after the reloading period. Concomitantly, repeated stimulation led to a significantly greater reduction in force production after disuse (Figure 3d), which remained unchanged after mechanical reloading.

### 3.4 Anatomical sex‐based differences in myofiber size are erased after disuse and reloading

**FIGURE 4:**
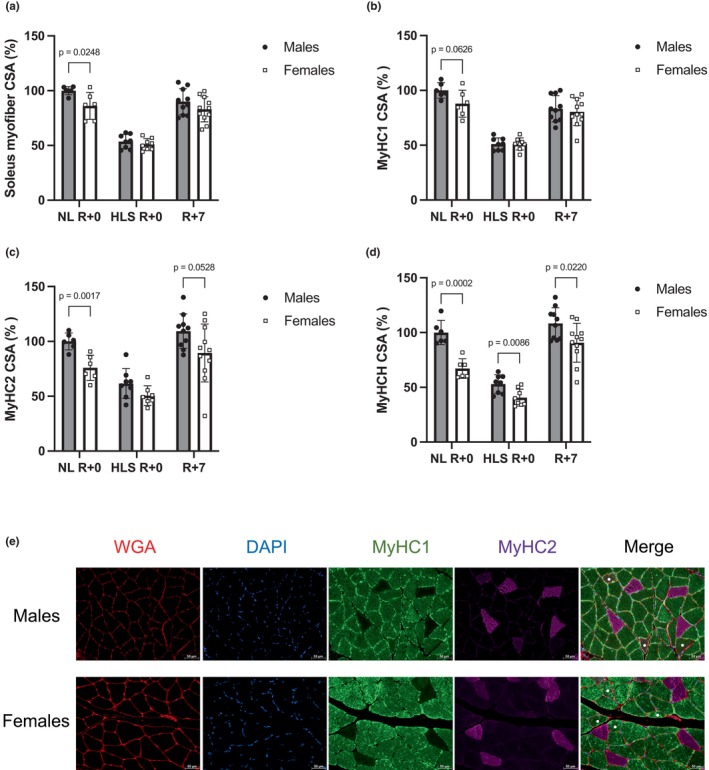
Soleus histomorphometry. Myofiber cross‐sectional area (CSA) compared to normally loaded males for mean CSA (a), slow‐twitch (MyHC1) fibers (b), fast‐twitch (MyHC2) fibers (c), and hybrid (MyHCH) myofibers (d), and representative images for males and females (e). Cell borders are shown in red (WGA); nuclei are represented in blue (DAPI); MyHC1 fibers are represented in green; MyHC2 fibers are represented in purple; and hybrid myofibers are labeled with white dots. Scale bar represents 50 μm. *N* = 10 for males, *n* = 11 for females. A table presenting the results of the two‐way ordinary ANOVA (factors of sex and time) and the results of the tests between males and females is displayed in Table S2. NL R + 0: animals that spent 14 days in normal loading conditions; HLS R + 0: animals that spent 14 days in hindlimb suspension; R + 7: animals that spent 14 days in hindlimb suspension and were reloaded for 7 days in normal loading conditions.

When normalized to body weight, to account for the large difference between males and females, hindlimb muscle mass did not significantly differ between sexes, despite females having overall larger relative muscle weights (Table 2). Histomorphometric analyses aimed to compare sex‐based differences in soleus myofiber cross‐sectional area (CSA) after reloading (R + 7) with disuse animals (HLS R + 0) and normally loaded controls (NL R + 0, Figure 4). Representative images are shown in Figure 4e. In normally loaded (NL) controls, females have 12%–33% smaller myofibers than males, depending on myofiber type. HLS results in myofiber atrophy in both males and females. Interestingly, after HLS, most of the sex‐based differences were no longer present with females having myofibers of similar size to males, with the exception of hybrid myofibers (MyHCH) that remained significantly smaller (*p* < 0.01, Figure 4d). Seven days of reloading at full weight‐bearing resulted in a significant increase in myofiber size in males and females across all fiber types. Sex‐based differences were not observed when analyzing average CSA nor in slow‐twitch (MyHC1) fibers (Figures 4a,b). These results emphasize that females had greater recovery of the MyHC1 soleus fibers than males, for which CSA remained significantly smaller than NL controls (*p* = 0.0064). Of note, after experiencing disuse and recovery, females but not males had larger MyHCH myofibers compared to their NL controls (*p* = 0.02, Figure 4d).

**TABLE 2:** Normalized hindlimb muscles wet mass.

Mass (g/100 g of body weight)	Soleus	Gastrocnemius	TA	EDL
Males (*n* = 10)	0.043 ± 0.005	0.553 ± 0.048	0.196 ± 0.015	0.044 ± 0.003
Females (*n* = 11)	0.047 ± 0.005	0.587 ± 0.068	0.205 ± 0.016	0.047 ± 0.003

*Note*: Results are displayed as mean ± SD and expressed in g/100 g of body weight and were obtained immediately at the end of the experiment (R + 7, Day 21). Results were analyzed using unpaired *t*‐tests. No significant differences were detected between males and females.

### 3.5 Estrous cycle is impacted by unloading and reloading in females

**FIGURE 5:**
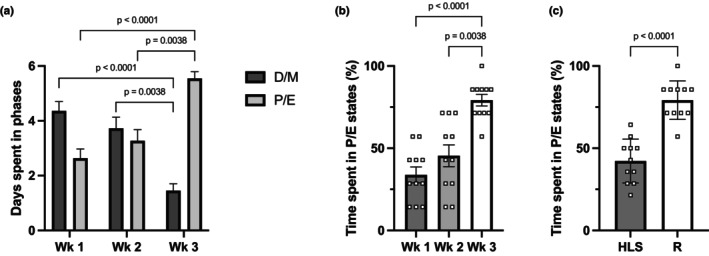
Estrous cycle analysis. Number of days spent in low hormone phases (Diestrus/Metestrus, D/M) and high hormone phases (Proestrus/Estrus, P/E) for each week of the protocol. Animals spent the first 2 weeks in hindlimb suspension and Week 3 at 1 g (a). Percentage of time spent in high hormone phases (P/E) for each week of the protocol (b), and for the full period of unloading (HLS, 14 days) and reloading (R, 7 days) (c). *N* = 11. Two‐way RM ANOVA was used for panel (a) with effects of time and estrus phase (time: *p* > 0.999, estrus: *p* = 0.0426, time × estrus: *p* < 0.0001). Results of the Tukey's post hoc tests are displayed and indicate a significant difference with the values obtained at Week 3. One‐way RM ANOVA was used for panel (b) (*p* < 0.0001), and results of the Tukey's post hoc tests are displayed. Paired Student's *t*‐test was used for panel (c).

Estrous cycle monitoring highlighted significant differences throughout the protocol (Figure 5). During the 2 weeks of unloading, females spent approximately 4 days per week in low hormone stages (i.e., Diestrus/Metestrus; D/M) and 3 days per week in high hormone stages (i.e., Proestrus/Estrus; P/E, Figure 5a). However, our animals experienced a significant shift during the week spent reloaded at 1 g, where they experienced 1.45 days in D/M and 5.55 days in P/E. This change in estrous cycle phases showed that during reloading at full weight‐bearing, our animals spent 79.2% of the time in high hormone stages, significantly more than during each week of HLS (Figure 5b), and almost twice as much as during the full unloading period (Figure 5c).

## 4 DISCUSSION

We studied the musculoskeletal effects of 7 days of recovery at full weight‐bearing following 14 days of mechanical unloading in adult male and female rats. Our results show that males experience greater trabecular bone loss during both the unloading and reloading periods compared to females and that females exhibit a greater recovery of muscle function. Moreover, following disuse and recovery, initial sex differences between males and females (e.g., such as myofibers being larger in healthy males compared to females) were eliminated, demonstrating that males experienced a greater loss and/or an incomplete recovery in the soleus muscle.

The most striking differences between males and females were observed in the skeletal compartment. Studies in rodents that underwent 4–39 days of spaceflight highlighted significant trabecular bone loss at an average rate of −1.7% per day (Stavnichuk et al., 2020) which is comparable to our findings in males. While the absence of bone loss in females may seem unexpected after 14 days of unloading, several teams have found similar results. Indeed, studies conducted in outbred rats showed no difference in tibial BMD, or femoral BMD after 2 to 3 weeks of hindlimb suspension (David et al., 2006; Lecoq et al., 2006; Peres‐Ueno et al., 2017) and observed only a 6.36% reduction in femoral BMD after 30 days of suspension (Lecoq et al., 2006). One study comparing bone adaptation after 2 weeks of unloading in male and female Wistar rats reported larger reductions in the cortical and cancellous compartments of males compared to females, due to increased bone resorption activity in males (David et al., 2006). Although the continued bone loss experienced by males after being allowed to freely ambulate for 7 days may seem surprising, human studies have found similar results, and highlighted that bone deterioration peaks after 15–30 days of re‐ambulation following bed rest in healthy men (Cervinka et al., 2014). Our results also showed increased cortical BMD during HLS for both sexes, with overall moderate changes compared to the trabecular compartment (maximum of 5% change from baseline), which has previously been reported in adult males exposed to partial weight‐bearing (Ko et al., 2020) and hindlimb unloading (Bloomfield et al., 2002) using in vivo pQCT, and further confirms the compartment‐specific behavior of the bone tissue (Bloomfield et al., 2002; Laib et al., 2000).

While few studies have directly compared the response of males and females to bouts of mechanical unloading, prior studies showed that female rats are more resistant to neuromuscular deconditioning than males when exposed to 14 days of HLS (Deschenes & Leathrum, 2016; Mortreux et al., 2021). However, in our current study, we did not observe differences regarding voluntary grip strength or fatigue resistance between sexes following disuse, similar to others (do Carmo et al., 2016). While these results are surprising given our earlier findings (Mortreux et al., 2021), the current study uses outbred animals with greater genetic variability to better emulate the individual variations found in the human population. Therefore, it is possible that some differences between our groups remained undetected. Although human studies have yet to reach a consensus regarding the impact of unloading on muscle function, multiple teams found consistent differences between men and women (Hicks et al., 2011). Indeed, force and endurance measurements performed in healthy participants (Clark et al., 2003; Fulco et al., 1999; Maughan et al., 1986; Russ & Kent‐Braun, 2003), and following limb immobilization (Semmler et al., 1999), repeatedly highlight that women have greater endurance capacity for isometric and dynamic muscle contractions compared to men. While functional decrements, as compared to simple force production, following unloading are sometimes observed to a greater extent in women (Deschenes et al., 2009; Wu et al., 2022; Yasuda et al., 2005), it is not always the case (de Carvalho et al., 2022; Mekjavic et al., 2021; Miles et al., 2005). In our animals, we observed greater improvement in muscle function after 7 days of recovery at full weight‐bearing in females than in males, and studies performed in humans have found similar results in participants recovering from immobilization (Semmler et al., 1999) and muscle exhaustion (Fulco et al., 1999). Finally, although rodent studies are heavily biased toward the exclusive use of males, several teams have reported decreased muscle function after reloading despite a recovery of muscle mass (Alway et al., 2014; Oliveira et al., 2019; Warren et al., 2004).

Sex dimorphism is observed in almost all tissues, including skeletal muscles (Glenmark et al., 2004; Haizlip et al., 2015; Miller et al., 1993), and is in part due to the presence of different sex hormones in males and females (Anderson et al., 2017), along with significant differences in gene expression (Colom et al., 2007). In both humans and rodents, males usually have larger muscles and myofibers (Deschenes et al., 2018; Mierzejewska‐Krzyżowska et al., 2019; Mortreux et al., 2021; Simard et al., 1987). They also have a greater proportion of slow‐twitch and hybrid fibers in weight‐bearing muscles such as the soleus (Novák et al., 2010). Previous work from our laboratory showed that males undergo more severe atrophy of the soleus and TA muscles than females during 14 days of hindlimb unloading (Mortreux et al., 2021) and others observed a significant loss of femoral bone density in males but not females, over the same period (David et al., 2006).

In our normally loaded animals, we observed that males had significantly larger myofibers than females, regardless of myofiber type. However, this sex dimorphism was largely absent in unloaded animals, except for hybrid myofibers (co‐expressing Myosin Heavy Chain types 1 and 2), that remained larger in males than females. Following 7d of reloading at normal weight‐bearing, myofiber size was not different between sexes, except for the hybrid fibers. These findings suggest that males suffer greater atrophy during disuse and a slower recovery during reloading compared to their female counterparts.

Estrous cycle rhythmicity could be a potential reason for these findings. Indeed, several studies have described the link between muscle atrophy and endocrine perturbations (Martín et al., 2018) and it has long been known that reductions in sex hormone levels (such as experienced after menopause or gonadectomy) are associated with greater musculoskeletal losses (Dupree & Dobs, 2004; Khadilkar, 2019; Navarrrete et al., 2020; Stratos et al., 2023). Recent experiments performed ex vivo and in vivo have shown that musculoskeletal deconditioning is directly correlated with the time spent in low‐hormonal phases (i.e., metestrus and diestrus) (Rosa‐Caldwell et al., 2021). In the current study, young adult females spent on average 57.8% of the time in low‐hormonal stages during HLS, similar to what has previously been reported (Rosa‐Caldwell et al., 2021). However, during the week of reloading, they spent only 20.8% of the time in low‐hormonal stages, which represents a significant shift that could have facilitated musculoskeletal recovery. These findings should be analyzed as those of young adult and skeletally mature animals, as rats experience hormonal changes throughout their life. As such, we cannot conclude that preservation of estrous cycle rhythmicity alone might be able to preserve musculoskeletal integrity during disuse in older animals. Further studies using 6‐month‐old animals and older are planned and will allow us to obtain a deeper understanding of the contribution of female sex hormones to musculoskeletal health.

One of the limitations of our study is that we did not track hormonal states in males. Although testosterone is a steroidal sex hormone known to be involved in musculoskeletal function (Florini, 1970; Herbst & Bhasin, 2004), the exact mechanisms at play remain elusive and numerous studies have described conflicting results (Brown, 2008; Gharahdaghi et al., 2021). Overall, researchers agree that testosterone acts as an anabolic molecule and increases muscle mass and strength, myofiber size, satellite cell activity (Serra et al., 2013) and promotes bone deposition. In parallel, low concentrations are linked with impaired muscle function (Ameredes et al., 1999; Grindeland et al., 1994) and low bone mineral density (Hind et al., 2006), and even observed in athletes suffering from Relative Energy Deficiency in Sports (RED‐S) (Mountjoy et al., 2014). Many studies reported that hindlimb unloading led to significant reductions in testosterone concentration (Karim et al., 2022; Mortreux et al., 2021; Moustafa, 2021; Ortiz et al., 1999); however, the impact on musculoskeletal deconditioning is not always evident. Some researchers have demonstrated that low testosterone did not amplify disuse‐induced muscle atrophy and loss of force (Brown et al., 2001; Rosa‐Caldwell et al., 1985), but others highlighted that low testosterone status attenuated musculoskeletal recovery during reloading (Hanson et al., 2020). In our study, we were expecting testosterone levels to drop in response to mechanical unloading as previously reported (De Naeyer et al., 2015), and it is possible that the exacerbated bone loss and attenuated muscle recovery during reloading may come from persistent low testosterone levels after re‐ambulation. This question awaits further study.

In summary, this study provides useful insight into the adaptations of the musculoskeletal system to full reloading following a period of unloading, mimicking a return to Earth after a prolonged stay in microgravity. We show that musculoskeletal recovery from unloading is sex‐dependent, with males experiencing a slower muscle recovery and more severe skeletal loss than females. Although disuse studies involving both sexes are infrequently performed and often do not collect concomitant hormonal data, our results demonstrate that such considerations are critical if we are to understand musculoskeletal alterations. Our results suggest that sex‐specific countermeasures and protocols could be needed to support future of space exploration and treat disuse‐related conditions here on Earth.

## AUTHOR CONTRIBUTIONS

MI, MRC, DS, and MM performed the experiments, MI, MRC, DS, and MM analyzed the results. MM prepared the figures. MI and MM drafted the manuscript. MM and SBR designed the research and experiments. All authors were involved in the revision of the manuscript and approved of its final version.

## FUNDING INFORMATION

This work was supported by a grant from the National Aeronautics and Space Administration (NASA: 80NSSC16K1598, S.B.R.; 80NSSC21K0311, S.B.R/M.R.C), a Scholarship FRIM from La Région Occitanie (M.I.), and internal funding from the College of Health Sciences at the University of Rhode Island (M.M.). The authors acknowledge the BIDMC Morphology Core for the use of their Histology and Epifluorescence Microscopy resources.

## CONFLICT OF INTEREST STATEMENT

The authors declare no conflicts of interests, financial, or otherwise.

## ETHICS STATEMENT

All experimental protocols were approved by the Beth Israel Deaconess Medical Center Institutional Animal Care and Use Committee under the protocol number #025‐2019.

## Supporting information


Figure S1.

Table S1.
**Table S2**.

## Data Availability

The data that support the findings of this study are available from the corresponding author upon reasonable request.
