# Correction to “Adaptation to full weight‐bearing following disuse in rats: The impact of biological sex on musculoskeletal recovery”

**DOI:** 10.14814/phy2.15978

**Published:** 2024-03-11

Issertine, M., Rosa‐Caldwell, M. E., Sung, D.‐M., Bouxsein, M. L., Rutkove, S. B., & Mortreux, M. (2024). Adaptation to full weight‐bearing following disuse in rats: The impact of biological sex on musculoskeletal recovery. *Physiological Reports*, *12*, DOI: 10.14814/phy2.15938.

In the above article, the second author's name was spelled incorrectly. The correct spelling is Megan E. Rosa‐Caldwell.

These errors have also been corrected online.

We apologize for this error.

