# Supplementary material for: Adaptation to full weight‐bearing following disuse in rats: The impact of biological sex on musculoskeletal recovery
Source: Physiol Rep. 2024 Feb 21;12(4):e15938. doi: 10.14814/phy2.15938 (PMC10881285; doi:10.14814/phy2.15938)
Supplement: Supplementary file 1 — Figure S1. [file PHY2-12-e15938-s001.pdf]

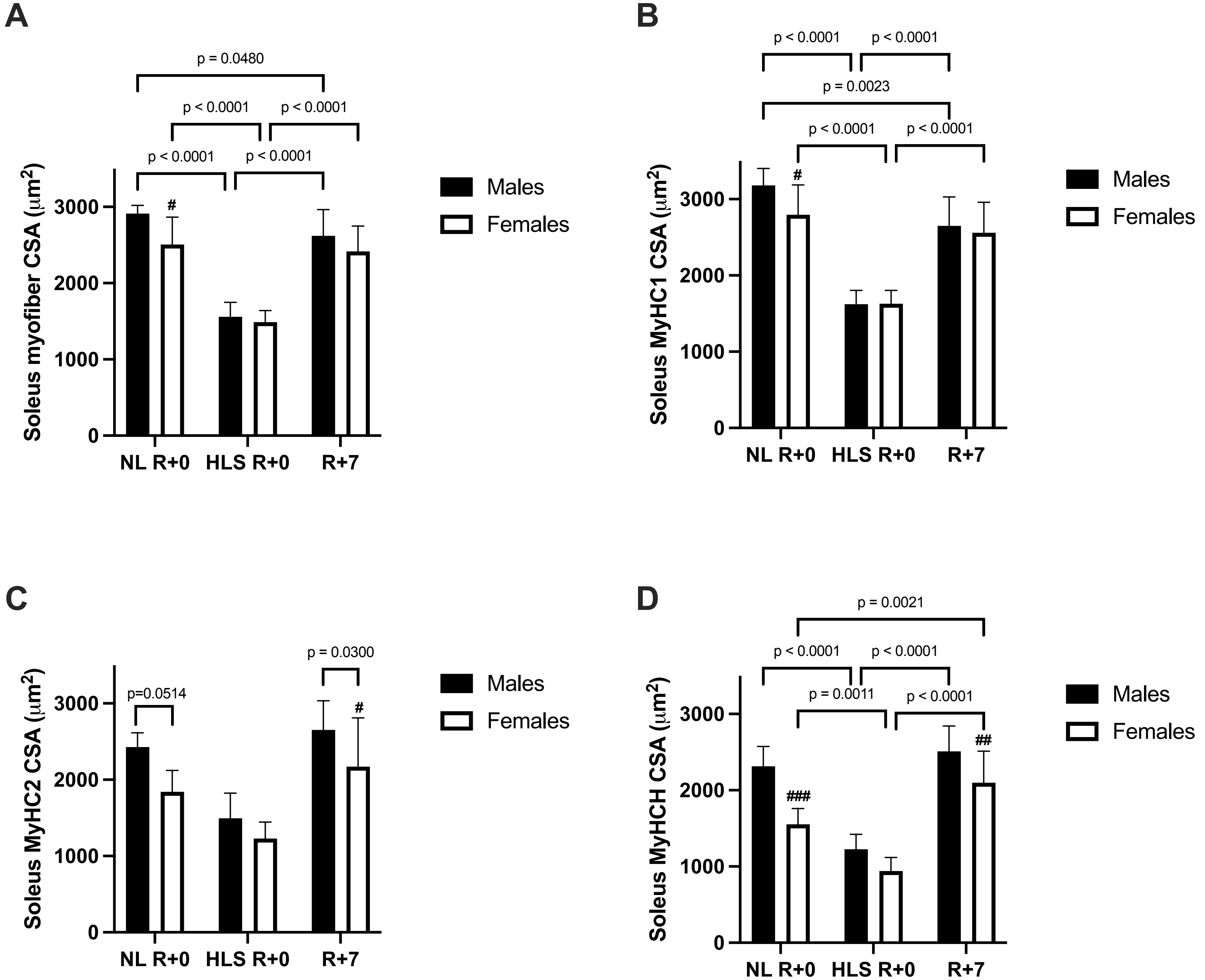

Supplementary Figure 1:

Histomorphometry of the soleus muscle in males and females exposed to 14 days of normal loading (NL R+0), 14 days of hindlimb suspension (HLS R+0), or 14 days of hindlimb suspension immediately followed by 7 days of recovery at normal loading (R+7).

Data from the NL R+0 and HLS R+0 animals has already been published (reference 32) in different arrangements.

N=6 per group for NL R+0, n=8 per group for HLS R+0, and n=10-11 per group for R+7.

Results were analyzed using 2-way ordinary ANOVA (factors of groups and sex), followed by Tukey's post hoc test. Group differences are represented by asterisks on the graph with *, **, ***, **** representing p<0.05, p<0.01, p<0.001, and p<0.0001, respectively. Differences between males and females at the same time point are represented as #, ##, ###: p<0.05, p<0.01, p<0.001, respectively.
