# Supplementary material for: Adaptation to full weight‐bearing following disuse in rats: The impact of biological sex on musculoskeletal recovery
Source: Physiol Rep. 2024 Feb 21;12(4):e15938. doi: 10.14814/phy2.15938 (PMC10881285; doi:10.14814/phy2.15938)
Supplement: Supplementary file 2 — Table S1. Table S2. [file PHY2-12-e15938-s002.docx]

**Supplementary Tables**

**Supplementary Table 1**: Statistical results for figure 1.

The results were analyzed in percentage of change from baseline and multiple comparisons were performed to compare the effects of time and sex using Sidak’s post hoc adjustments.

|  | **Main Effect** | | **2-way** | **Multiple comparison Males** | | | **Multiple comparison Females** | | | **Multiple comparisons**  **Males vs Females** | |
| --- | --- | --- | --- | --- | --- | --- | --- | --- | --- | --- | --- |
| **Panel** | **Time** | **Sex** | **Time * Sex** | **Day 0 vs Day 14** | **Day 0 vs Day 21** | **Day 14 vs Day 21** | **Day 0 vs Day 14** | **Day 0 vs Day 21** | **Day 14 vs Day 21** | **Day 14** | **Day 21** |
| **1A** | <0.0001 | <0.0001 | <0.0001 | <0.0001 | <0.0001 | 0.0415 | 0.0668 | 0.0923 | 0.9998 | 0.0037 | <0.0001 |
| **1B** | <0.0001 | 0.7139 | 0.1672 | 0.0026 | 0.0084 | 0.0195 | 0.0001 | <0.0001 | 0.7478 | 0.6806 | 0.8927 |
| **2A** | 0.0005 | 0.2493 | 0.1114 | >0.9999 | 0.3610 | 0.0895 | 0.8858 | 0.0121 | <0.0001 | 0.9415 | 0.2729 |
| **2B** | <0.0001 | 0.0864 | 0.0592 | 0.0004 | 0.0409 | 0.0384 | <0.0001 | 0.8797 | <0.0001 | 0.9991 | 0.0464 |
| **2C** | 0.1215 | 0.8251 | 0.1676 | 0.9931 | 0.9790 | 0.9836 | 0.0695 | 0.3141 | 0.0007 | 0.4494 | 0.9170 |
| **2D** | 0.4444 | 0.0461 | 0.0205 | 0.9693 | 0.2090 | 0.4255 | 0.4983 | 0.2776 | 0.4670 | 0.4640 | 0.0994 |
| **3A** | 0.1161 | 0.5442 | 0.7818 | 0.9202 | 0.6457 | 0.5193 | 0.4382 | 0.8991 | 0.0651 | 0.7780 | 0.9795 |
| **3B** | 0.0013 | 0.2153 | 0.2940 | 0.0366 | 0.0787 | 0.9849 | 0.6132 | 0.1306 | 0.1033 | 0.3961 | 0.7387 |
| **3C** | <0.0001 | 0.4326 | 0.6024 | 0.0058 | 0.0199 | 0.4309 | 0.0140 | 0.2359 | 0.0505 | 0.8326 | 0.8323 |
| **3D** | 0.0031 | 0.5518 | 0.7469 | 0.1477 | 0.1674 | 0.8554 | 0.0474 | 0.3843 | 0.7187 | 0.9338 | 0.9114 |

**Supplementary Table 2**: Statistical results for figure 4

We performed a 2-way ordinary ANOVA (factors of sex and time) and indicated the results of the post-hoc tests in males and females.

|  | **Main Effects** | | **2-way** | **Multiple comparisons Males** | | | **Multiple comparisons Females** | | |
| --- | --- | --- | --- | --- | --- | --- | --- | --- | --- |
| **Panel** | **Time** | **Sex** | **Time * Sex** | **NL R+0 vs HLS R+0** | **NL R+0 vs R+7** | **HLS R+0 vs R+7** | **NL R+0 vs HLS R+0** | **NL R+0 vs R+7** | **HLS R+0 vs R+7** |
| **4A** | <0.0001 | <0.0001 | <0.0001 | <0.0001 | 0.1374 | <0.0001 | <0.0001 | 0.8915 | <0.0001 |
| **4B** | <0.0001 | 0.6319 | 0.2964 | <0.0001 | 0.0064 | <0.0001 | <0.0001 | 0.3171 | <0.0001 |
| **4C** | <0.0001 | 0.0283 | 0.2481 | 0.0004 | 0.5390 | <0.0001 | 0.0268 | 0.2643 | <0.0001 |
| **4D** | 0.0087 | 0.0614 | 0.4069 | <0.0001 | 0.4161 | <0.0001 | 0.0010 | 0.0020 | <0.0001 |
